# Development and Validation of Survival Nomograms in Patients with Primary Bladder Lymphoma

**DOI:** 10.3390/jcm11113188

**Published:** 2022-06-02

**Authors:** Junyi Lin, Jianbin Kong, Mingli Luo, Zefeng Shen, Shuogui Fang, Jintao Hu, Zixin Xu, Wen Dong, Jian Huang, Tianxin Lin

**Affiliations:** 1 Department of Urology, Sun Yat-sen Memorial Hospital, Sun Yat-sen University, Guangzhou 510120, China; linjy23@mail2.sysu.edu.cn (J.L.); luomli3@mail2.sysu.edu.cn (M.L.); shenzf@mail2.sysu.edu.cn (Z.S.); hujt7@mail2.sysu.edu.cn (J.H.); xuzx27@mail2.sysu.edu.cn (Z.X.); dongwen@mail.sysu.edu.cn (W.D.); 2 Guangdong Provincial Key Laboratory of Malignant Tumor Epigenetics and Gene Regulation, Sun Yat-sen Memorial Hospital, Sun Yat-sen University, Guangzhou 510120, China; 3 School of Medicine, Shenzhen Campus of Sun Yat-sen University, Sun Yat-sen University, Shenzhen 518107, China; kongjb@mail2.sysu.edu.cn; 4 Department of Thoracic Surgery, Sun Yat-sen University Cancer Center, Guangzhou 510060, China; fangsg@sysucc.org.cn

**Keywords:** primary bladder lymphoma, overall survival, cancer-specific survival, nomogram, Surveillance, Epidemiology, and End Results (SEER)

## Abstract

Background: The existing studies on primary bladder lymphoma (PBL) are retrospective analyses based on individual cases or small series studies, and the research on PBL is not unified and in-depth enough at present because of the scarcity of PBL and the lack of relevant literature. This study is designed to develop and validate nomograms for overall survival (OS) and cancer-specific survival (CSS) prediction in patients with PBL. Methods: According to the Surveillance, Epidemiology, and End Results (SEER) database, 405 patients diagnosed with PBL from 1975 to 2016 were collected and randomly assigned to training (*n* = 283) and validation (*n* = 122) cohort. After the multivariable Cox regression, the OS and CSS nomograms were developed. The discrimination, calibration and clinical usefulness of the nomograms were assessed and validated, respectively, by the training and validation cohort. Furthermore, all of the patients were reclassified into high- and low-risk groups and their survival were compared through Kaplan-Meier method and log-rank test. Results: Age, subtype, Ann Arbor stage, radiation and chemotherapy were identified as independent prognostic factors for OS and age, sex, and subtype for CSS, then corresponding nomograms predicting the 3- and 5-year survival were constructed. The presented nomograms demonstrated good discrimination and calibration, which the C-index in the training and validation cohort were 0.744 (95% confidence interval [CI], 0.705–0.783) and 0.675 (95% CI, 0.603–0.747) for OS nomogram and 0.692 (95% CI, 0.632–0.752) and 0.646 (95% CI, 0.549–0.743) for CSS nomogram, respectively. Furthermore, the nomograms can be used to effectively distinguish Patients with PBL at high risk of death. The clinical usefulness of the nomograms was visually displayed by decision curve analysis. Conclusion: We updated the baseline characteristics of patients with PBL and constructed OS and CSS nomograms to predict their 3- and 5-year survival. Using these nomograms, it would be convenient to individually predict the prognosis of patients with PBL and provide guidance for clinical treatment.

## 1. Introduction

Bladder lymphoma can be categorized into primary bladder lymphoma (PBL) and secondary bladder lymphoma. The former originates in the urinary bladder, without lymphoma found in other tissues of the body, and the latter is related to lymphoma originating from other tissues outside the bladder [1,2,3,4]. PBL cases have been reported since 1885, and as a relatively rare disease, it accounts for only 0.2% of extranodal lymphomas (25% to 40% of all lymphomas) and less than 1% of all primary bladder tumors [2,5,6,7].

PBL tends to develop in middle-aged females, with a reported male-to-female ratio of 1:3 by Al-Maghrabi et al. to 1:6.5 by Ohsawa et al., and most patients are diagnosed in their sixties [8,9,10]. The most common and obvious clinical symptom of PBL is gross hematuria, and symptoms such as urinary frequency, dysuria, abdominal pain, and weight loss may also occur [8,11,12]. The imaging findings of bladder lymphoma were submucosal masses, of which 70% were solitary mass, 20% were multiple masses and 10% were diffuse thickening of the bladder wall [4]. Most PBL are low-grade tumors, while high-grade tumors only account for 20% [2]. The most common type among low-grade PBL is extranodal marginal zone/mucosa-associated lymphoid tissue (MALT) lymphoma, accounting for 86% of low-grade cases and 53% of all cases [5,10,13], and the most common type among high-grade PBL is diffuse large B-cell lymphoma (DLBCL) [12]. Currently, there are no standardized protocols or guidelines for the treatment of PBL. Partial or radical cystectomy, radiotherapy or chemotherapy, or comprehensive therapy have been reported to treat PBL, and according to Hughes et al., no matter which treatment is taken, the complete remission rates of Patients with PBL approach nearly 100% [4,13].

It has been reported that PBL has a generally good prognosis, with a relatively long-median survival time and slow disease progression [5,12]. Factors reported to be associated with PBL prognosis include age [14], histological subtype [4], grade and stage of the tumor [4,15], etc. However, the existing studies on PBL are retrospective analyses based on individual cases or small series studies, and the research on PBL is not unified and in-depth enough at present because of the scarcity of PBL and the lack of relevant literature. Hence, we attempted to explore the baseline characteristics and prognostic factors of PBL by using the data of the Surveillance, Epidemiology, and End Results (SEER) database, and then established prognostic nomogram in order to help clinicians accurately estimate the prognosis and to provide guidance and assistance for clinical treatment.

## 2. Materials and Methods

### 2.1. Patients and Data Collection

Information about patients who were diagnosed with primary bladder lymphoma from SEER database (https://seer.cancer.gov/, accession number: 15751-Nov2020, accessed on 21 January 2022.) were collected through SEER*Stat software. The inclusion criteria were: (1) diagnosis of lymphoma; and (2) the primary site was urinary bladder. The following were exclusion criteria: (1) without complete demographic or clinicopathological information or follow-up information; (2) if the primary site of the tumor was confined to the ureteral orifice, for the reason that only one record was not allowed to be grouped. Specially, we used the AYA site recode/WHO 2008 code 2 lymphomas and primary site codes C67.0–C67.9 to identify lymphoma that primarily found in the bladder, and finally, 405 patients who had received a diagnosis between 1975 and 2016 were included. The following clinicopathological information were of concern: age, sex, race, marital status, site, subtype, Ann Arbor stage, and three therapy methods, including surgery, radiotherapy, and chemotherapy, etc. The Ann Arbor stage system is the classification criteria for lymphoma, and stage I to IV refers, respectively, to (I) one group of adjacent nodes involvement or single extranodal lesions without nodal involvement, (II) two or more nodal groups on the same side of the diaphragm or limited contiguous extranodal involvement, (III) nodes on both sides of the diaphragm involvement or nodes above the diaphragm with spleen involvement, (IV) additional noncontiguous extralymphatic involvement [16]. Overall survival (OS) is defined as the time from diagnosis to death regardless of cause and cancer-specific survival (CSS) is defined as the time from diagnosis until death because of PBL or end of follow-up, which are the primary outcome of the study.

**Figure 1:**
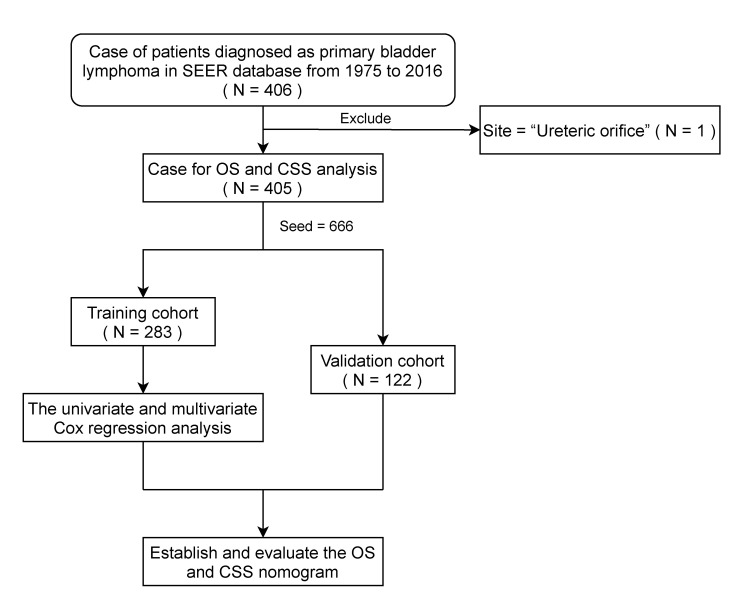
Flowchart of case selection and analysis process of patients diagnosed as primary bladder lymphoma from SEER database. SEER, Surveillance, Epidemiology, and End Result; OS, overall survival; CSS, cancer-specific survival.

After screening by the above methods, all eligible PBL randomly assigned to training and validation cohort in proportion of 7:3. Figure 1 shows the flowchart of the study.

### 2.2. Model Development

The univariable and multivariable Cox regression algorithm were performed in the training cohort to analyze clinicopathological predictors for OS and CSS, and significant predictors were selected according to backward stepwise selection. Finally, nomograms predicting OS and CSS at the 3- and 5-year stage were developed [17,18].

### 2.3. Performance Assessment and Validation of the Model

The nomograms’ performance was assessed through the Harrell’s concordance index (C-index), calibration curves and the area under the curve (AUC) of the receiver operator characteristic (ROC). Discriminative ability was quantitatively assessed by the C-index [18], and calibration by calibration curves. It was worth mentioning that the C-index was acquired by bootstrapping with 1000 resampling procedures. The nomogram multivariable Cox regression formula was implemented to the validation cohort, and the same performance evaluation was performed in the validation cohort to validate the performance.

### 2.4. Survival Risk Classification Based on the Nomogram

According to the formula from multivariable Cox regression, all of the patients’ risk scores were+ calculated.

Risk score = a_1_F_1_ + a_2_F_2_ + … + a_i_F_i_

where a_i_ and F_i_ represent the selected survival-related risk factors and the associated coefficient, respectively.

The X-tile software was used to obtain the optimal risk score cutoff value [19], and according to the cutoff value, patients with PBL were reclassified into high- and low-risk groups. The differences of OS and CSS between the two groups were demonstrated through Kaplan-Meier method and log-rank test.

### 2.5. Clinical Usefulness of the Nomogram

Decision curve analysis (DCA) was carried out to assess the nomogram’s clinical usefulness, which is a method for estimating the clinical benefit of the model, using the curves of full treatment regimen curves and no treatment regimen as two references, and the net benefit is quantified at different threshold probabilities of the nomogram [20].

### 2.6. Statistical Analysis

We used the X-tile software version 3.6.1 (Yale University School of Medicine, New Haven, CT, USA) to carry out X-tile plots for determining the optimal cutoff value of risk score, and R statistical software, version 4.1.1 (The R Foundation for Statistical Computing, https://www.r-project.org/, accessed on 21 January 2022.) to all other statistical analyses.

The Cox regression model, nomogram and calibration curve, ROC curve was completed by the R packages “survival” and “MASS”, “rms”, “survivalROC”, respectively. The function “stdca.R” was used to perform DCA. Continuous data and categorical variables were shown as median [interquartile range (IQR)] and number (percentage), respectively. *p*-value < 0.05 (2-sided) was the standard of statistically significant.

## 3. Results

### 3.1. Baseline Characteristics of Total Patients with PBL

A total of 406 patients diagnosed with PBL were collected in SEER database from year 1975 to 2016, and 1 patient met the exclusion criteria, so eventually 405 patients were included. These 405 patients were then randomly assigned to training (*n* = 283) and validation (*n* = 122) cohort according to the ratio of 7:3 (Table 1). The median age of patients with PBL was 76.00 years old (interquartile ranges [IQR], 64.00–84.00 years old), with an approximate 2:3 male to female ratio. Most of patients were white people (*n* = 342, 84.4%), with black people (*n* = 23) and other/unknown races (*n* = 40) accounting for 5.7% and 9.9%, respectively. The sites of most lymphoma in the bladder were not otherwise specified (NOS). The subtypes were as follows: 50.1%, aggressive B cell non-Hodgkin’s lymphoma (NHL); 30.4%, indolent B cell NHL; 11.6%, NHL-NOS; and 7.9%, Other/unclassified. Patients at Ann Arbor stage I accounted for the majority (*n* = 213, 52.6%), while 15.1%, 4.2%, 16.8% for patients at stage II, III, IV, respectively. Over 75% (*n* = 317) of the patients received radiation, while only about half underwent surgery (48.6%) or chemotherapy (52.6%). During follow-up, 262 patients (64.7%) dead among all 405 patients involved, and the median follow-up time for total patients with PBL was 31 months (IQR, 5–92 months).

### 3.2. Model Development

**Figure 2:**
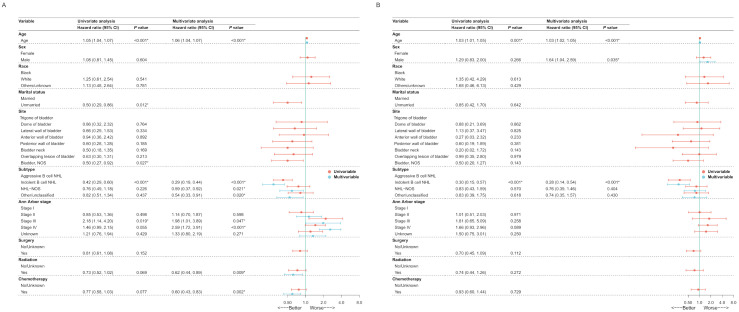
The Univariate and Multivariate Cox regression analysis in training cohort (N = 283) of patients with primary bladder lymphoma for OS (**A**) and CSS (**B**). OS, overall survival; CSS, cancer-specific survival. * *p* < 0.05.

**Figure 3:**
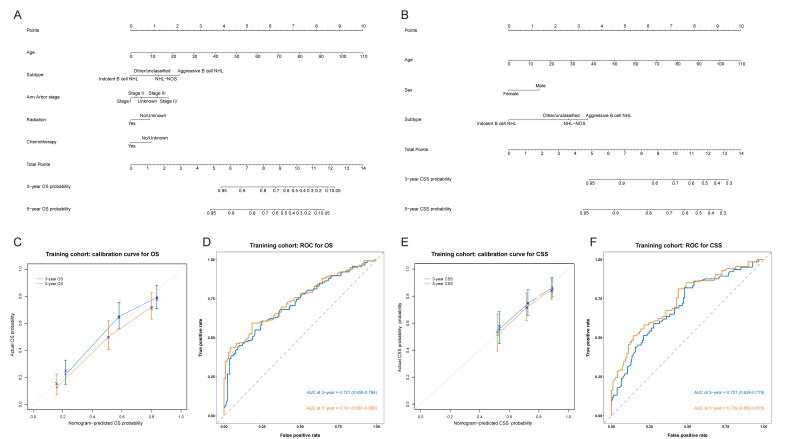
Nomograms to predict 3-year and 5-year OS (**A**) and CSS (**B**) of patients with primary bladder lymphoma, and calibration curves and ROC of the OS nomogram (**C**,**D**) and CSS nomogram (**E**,**F**) in the training cohort. The AUC at 3-year (the blue line) and 5-year (the orange line) was 0.721(95% CI, 0.658–0.784) and 0.741(95% CI, 0.681–0.802) for the OS nomogram, and 0.701 (95% CI, 0.624–0.779) and 0.739 (95% CI, 0.662–0.816) for the CSS nomogram. NHL, non-Hodgkin’s lymphoma; NOS, not otherwise specified; OS, overall survival; CSS, cancer-specific survival; ROC, receiver operating characteristic curve; AUC, areas under the curve.

The Cox regression analysis was performed on 10 potential variables, age, sex, race, marital status, site, subtype, Ann Arbor stage, surgery, radiation and chemotherapy, in the training cohort, for the purpose of determining independent prognostic factors for survival of PBL, and Figure 2A,B demonstrates the result of the Cox regression analysis for OS and CSS, respectively. Finally, age, subtype, Ann Arbor stage, radiation and chemotherapy were identified as independent prognostic factors for OS and age, sex, and subtype for CSS. Then 2 prediction models were built based on these factors, which were visualized via nomogram (Figure 3A,B) to predict patients with PBL’ 3- and 5-year survival.

### 3.3. Performance Assessment and Validation of The Nomogram

The C-index in the training and validation cohort of OS nomogram was 0.744 (95% CI, 0.705–0.783) and 0. 675 (95% CI, 0.603–0.747), respectively, and 0.692 (95% CI, 0.632–0.752) and 0. 646 (95% CI, 0.549–0.743) of CSS nomogram, showing considerably good discrimination of newly established nomograms. Calibration curves in the training cohort for OS nomogram (Figure 3C) and CSS nomogram (Figure 3E) separately demonstrated a great consistency between actual and predicted survival, analogously, calibration curves (Supplementary Figure S1) in the validation cohort and all patients demonstrated the consistent pattern.

According to the nomogram, ROC curves were drawn to verify ability in order to accurately predict survival. In the training cohort, the 3- and 5-year AUC of OS nomogram’s ROC curve was 0.721 (95% CI, 0.658–0.784) and 0.741 (95%CI, 0.681–0.802) (Figure 3D), and 0.701 (95% CI, 0.624–0.779) and 0.739 (95% CI, 0.662–0.816) of CSS nomogram’s ROC curve (Figure 3F), showing good sensitivity and specificity of the nomogram. At the same time, as shown in Supplementary Figure S2, for OS nomogram, the 3- and 5-year AUC in the validation cohort was 0.667 (95% CI, 0.566–0.769) and 0.653 (95% CI, 0.546–0.760), and 0.708 (95% CI, 0.654–0.761) and 0.717 (95% CI, 0.664–0.770) in all patients; for CSS nomogram, the the 3- and 5-year AUC in the validation cohort was 0.699 (95% CI, 0.577–0.821) and 0.611 (95% CI, 0.467–0.754), and 0.698 (95% CI, 0.633–0.764) and 0.701 (95% CI, 0.632–0.770) in all of the patients. Therefore, both nomograms performed well.

### 3.4. Survival Risk Classification Based on the Nomogram and Prognosis Analysis

The nomogram’s Cox regression coefficients and risk score’s calculating formula for OS nomogram were provided in Supplementary Tables S1 and S2, and for CSS nomogram in Supplementary Tables S3 and S4. After calculating the patient’s final score by summing up each element in the nomogram, the optimal cut-off value was set to be 11.14 for the OS risk score and 11.62 for the CSS risk score in patients with PBL with the help of X-tile software (Supplementary Figure S3).

**Figure 4:**
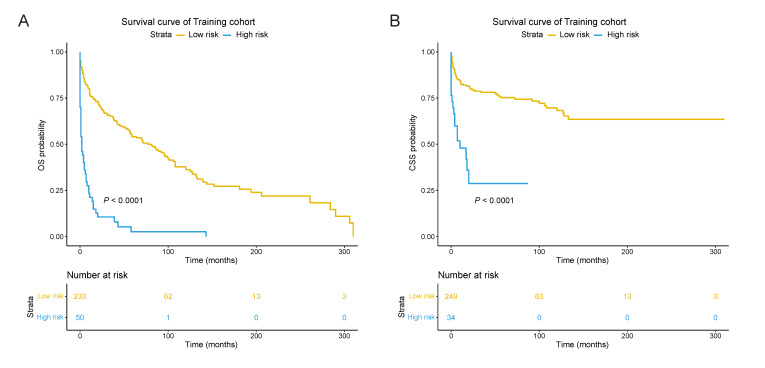
The OS (**A**) and CSS (**B**) Kaplan-Meier curves between high- and low-risk group of patients with primary bladder lymphoma in the training cohort. High-risk of OS: nomogram score ≥ 11.14; Low-risk of OS: nomogram score < 11.14; High-risk of OS: nomogram score ≥ 11.62; Low-risk of CSS: nomogram score < 11.62; OS, overall survival; CSS, cancer-specific survival.

Patients with a OS nomogram risk score ≥ 11.14 and a CSS nomogram risk score ≥ 11.62 were reclassified into OS high-risk group and CSS high-risk group, respectively, while the other into corresponding low-risk group. The Kaplan-Meier curves in the training cohort indicated worse OS and CSS in high-risk group with statistically significant difference (*p* < 0.0001, Figure 4). Analogously, the same association also appeared in the validation cohort and all of the patients for either OS (*p* < 0.0001) or CSS (*p* = 0.029 and *p* < 0.0001) (Supplementary Figure S4). The results indicated that the 2 nomograms can be used to effectively distinguish patients with PBL at high risk of death. Among 405 patients involved, the median survival time for the high- and low-risk group was 44 months (IQR, 10–101 months) 2 months (IQR, 1–10 months) when it comes to OS, and 42 months (IQR, 6–100 months) and 6 months (IQR, 1–15 months) when it comes to CSS.

### 3.5. Clinical Usefulness of the Nomogram

DCA were demonstrated in Supplementary Figure S5 to visualize the net benefit at the 3- and 5-year stage. The results of the DCA indicated that using nomogram to predict survival and make treatment decisions would be more beneficial than either the full treatment regimen or the no treatment regimen. Therefore, the nomograms constructed in the study is clinically useful.

## 4. Discussion

From the SEER database, we included 405 patients to describe and analyze the characteristics of PBL and then build prognostic prediction models. Age, subtype, Ann Arbor stage, radiation and chemotherapy were eventually identified to predict OS, and age, sex and subtype were eventually identified to predict CSS. The nomograms had a good discrimination, calibration, and clinical usefulness. Therefore, the established PBL prediction models play an auxiliary role in explaining the characteristics and making clinical decisions for patients with PBL.

In our study, the median age of patients with PBL was 76 years, which was older than the previously reported 64 years by Ohsawa et al. [8]. Given that the sample size included in this study was more than 100 times that of previous studies, we believe that the average age of onset we obtained this time is more convincing. The results of the analysis also revealed a negative correlation between age and survival. Consistent with previous reports, the prevalence of PBL in women is higher than that in men in our study. However, the male-to-female ratio of PBL was 2:3, which was different from the values previously reported by Al-Maghrabi et al. and Ohsawa et al. [8,10]. We still believe that our study calculated the ratio more accurately due to our larger sample size. Interestingly, past research has shown that estrogen can inhibit lymphoma in female patients by reducing serum IL-6, so the incidence of lymphoma in women is generally lower in women than in men [21,22]. However, PBL has an obvious tendency to occur in women, and the mechanism remains to be studied.

Khaitan et al. and Fernández et al. found that chronic cystitis was often presented as the baseline characteristic in patients with PBL, and about 20% of cases were confirmed to have a history of chronic cystitis [5,23]. Considering that the bladder is an organ lacking naturally occurring lymphoid tissue, cystitis is thought to be a possible cause of PBL [2,13]. However, cystitis is a common disease and PBL is rare. Therefore, some researchers believe that cystitis may only play a part in the complex process of PBL formation [14]. If this hypothesis is confirmed, it may explain why PBL tends to occur in women, because the unique anatomical structure and physiological characteristics of women (such as short urethra, urethra near the vagina and anus, menstruation, etc.) lead to their susceptibility to urinary tract infections. Unfortunately, the item of cystitis is not included in the SEER database, so this idea is unable to be verified currently and further research is needed.

As an important prediction model, nomograms have been extensively used in prognostic research [24]. In this study, age, subtype, Ann Arbor stage, radiation and chemotherapy were identified as independent prognostic factors for OS in patients with PBL, age, sex and subtype for CSS, then corresponding OS and CSS nomogram predicting the 3- and 5-year survival were constructed. In order to check whether the constructed prediction model is over-fitting or under-fitting, and to make it generally applicable and persuasive, the validation of nomograms’ performance is necessary. In this study, all assessments indicated that the nomogram could precisely predict the survival and make treatment decisions more beneficial. Ground on the nomogram, clinicians can predict the outcome of a PBL patient and make subsequent treatment plans accordingly.

Although PBL is reported to have a generally good prognosis, there are still a small number of patients with PBL with a poor prognosis, for whom it is difficult to achieve long-term survival after treatment. In order to detect these patients with PBL with potentially poor prognosis in time, we reasonably and innovatively proposed to reclassify patients with PBL into high- and low-risk group with OS nomogram score of 11.14 and CSS nomogram score of 11.62 as the cut-off value and confirmed a worse survival of high-risk group. Therefore, if the nomogram identifies a patient whose OS score is greater than 11.14, or CSS score is greater than 11.62, the clinician can be vigilant and adjust the follow-up treatment methods in time, such as using combined modality therapy as soon as possible, in order to make the patient obtain better curative effect. Under the circumstances, the nomogram plays a great role.

There are inevitably some limitations to this study. First, we collected information from a single database for retrospective analysis, therefore, whether the nomogram can be extended to the prognostic analysis of all patients with PBL remains to be verified. It should be emphasized that some prospective external validations are needed before ap-plying the nomogram in clinical practice. Secondly, because the existing literature was insufficient and the items owned by SEER data were limited, some potential prognostic factors, such as cytokines and disease complications, might not be found by us. Thirdly, among the patients with PBL we enrolled, the time interval between the earliest data and the latest data was quite long, during which the detection and treatment techniques may change and may affect the survival and status of patients. Finally, limited by too few cases of hodgkin lymphoma of PBL in the SEER database, we failed to classify them separately, so we also hope to refine bladder lymphoma into Hodgkin’s lymphoma and non-Hodgkin’s lymphoma in the future, thus, its prognosis can be more accurately predicted. Although these limitations exist, it is still the first study developing a nomogram with the largest number of PBL cases so far, so the results of our analysis can still provide important help for the research and treatment of PBL. We hope that the follow-up studies can optimize this prediction model.

## 5. Conclusions

In conclusion, we updated the baseline characteristics of patients with PBL and constructed OS and CSS nomograms to predict their 3- and 5-year survival. These nomograms and the corresponding risk classification system can help clinicians estimate the prognosis accurately and provide guidance for clinical treatment.

## Figures and Tables

**Table 1:** Baseline characteristics of original data set patients with primary bladder lymphoma (N = 405).

	Training Cohort, N = 283	Validation Cohort, N = 122	*p* Value
**Age (median [IQR])**	75.00 [64.50, 83.00]	77.00 [64.00, 84.00]	0.727
**Sex**			0.464
Female	175 (61.84%)	70 (57.38%)	
Male	108 (38.16%)	52 (42.62%)	
**Race**			0.236
White	237 (83.75%)	105 (86.06%)	
Black	14 (4.95%)	9 (7.38%)	
Other/Unknown	32 (11.30%)	8 (6.56%)	
**Marital status**			1.000
Married/Unknown	250 (88.34%)	108 (88.52%)	
Unmarried	33 (11.66%)	14 (11.48%)	
**Site**			0.231
Trigone of bladder	14 (4.95%)	4 (3.28%)	
Dome of bladder	8 (2.83%)	6 (4.92%)	
Lateral wall of bladder	15 (5.30%)	11 (9.02%)	
Anterior wall of bladder	9 (3.18%)	3 (2.46%)	
Posterior wall of bladder	22 (7.77%)	15 (12.29%)	
Bladder neck	10 (3.53%)	6 (4.92%)	
Overlapping lesion of bladder	27 (9.54%)	5 (4.10%)	
Bladder, NOS	178 (62.90%)	72 (59.01%)	
**Subtype**			0.324
Aggressive B cell NHL	144 (50.88%)	59 (48.36%)	
Indolent B cell NHL	79 (27.92%)	44 (36.06%)	
NHL-NOS	35 (12.37%)	12 (9.84%)	
Other/unclassified	25 (8.83%)	7 (5.74%)	
**Ann Arbor stage**			0.828
Stage I	147 (51.94%)	66 (54.10%)	
Stage II	41 (14.49%)	20 (16.39%)	
Stage III	11 (3.89%)	6 (4.92%)	
Stage IV	49 (17.31%)	19 (15.57%)	
Unknown	35 (12.37%)	11 (9.02%)	
**Surgery**			
Yes	139 (49.12%)	58 (47.54%)	0.855
No/Unknown	144 (50.88%)	64 (52.6%)	
**Radiation**			
Yes	215 (75.97%)	102 (83.61%)	0.115
No/Unknown	68 (24.03%)	20 (16.39%)	
**Chemotherapy**			
Yes	150 (53.00%)	63 (51.64%)	0.886
No/Unknown	133 (47.00%)	59 (48.36%)	
**Follow-up time (median [IQR])**	27.00 [5.00, 91.00]	37.00 [6.00, 93.75]	0.726

IQR, interquartile range; NOS, not otherwise specified; NHL, non-Hodgkin’s lymphoma.

## Data Availability

Publicly available datasets were analyzed in this study. This data can be found here: https://seer.cancer.gov/, accessed on 21 January 2022.

## Supplementary Materials

The following supporting information can be downloaded at: https://www.mdpi.com/article/10.3390/jcm11113188/s1, Figure S1. Calibration curve in the validation cohort and all patients of the OS nomogram (A,B) and CSS nomogram (C,D); Figure S2. The ROC in the validation cohort (A) and in all patients (B) of the OS nomogram and in the validation cohort (C) and in all patients (D) of the CSS nomogram; Figure S3. The optimal cut-off value calculated by X-tile software was 11.14 for the OS nomogram score and 11.62 for the CSS nomogram score; Figure S4. The Kaplan-Meier curves in the validation cohort (A) and in all patients (B) of the OS nomogram and in the validation cohort (C) and in all patients (D) of the CSS nomogram for patients with primary bladder lymphoma in the high- and low-risk group; Figure S5. DCA for OS in the training cohort (A,B), validation cohort (C,D) and in all patients (E,F), and for CSS in the training cohort (G,H), validation cohort (I,J) and in all patients (K,L); Table S1. The Cox regression coefficients of the OS nomogram; Table S2. The Cox regression coefficients of the CSS nomogram; Table S3. The formula of each variable to calculate the OS risk score; Table S4. The formula of each variable to calculate the CSS risk score.

## References

[1] Evans D.A., Moore A.T. (2007). The first case of vesico-vaginal fistula in a patient with primary lymphoma of the bladder—A case report. J. Med. Case Rep..

[2] Maninderpal K.G., Amir F.H., Azad H.A., Mun K.S. (2011). Imaging findings of a primary bladder maltoma. Br. J. Radiol..

[3] Matsuda I., Zozumi M., Tsuchida Y.A., Kimura N., Liu N.N., Fujimori Y., Okada M., Hashimoto T., Yamamoto S., Hirota S. (2014). Primary extranodal marginal zone lymphoma of mucosa-associated lymphoid tissue type with malakoplakia in the urinary bladder: A case report. Int. J. Clin. Exp. Pathol..

[4] Venyo A.K. (2014). Lymphoma of the urinary bladder. Adv. Urol..

[5] Kempton C.L., Kurtin P.J., Inwards D.J., Wollan P., Bostwick D.G. (1997). Malignant lymphoma of the bladder: Evidence from 36 cases that low-grade lymphoma of the MALT-type is the most common primary bladder lymphoma. Am. J. Surg. Pathol..

[6] Mourad W.A., Khalil S., Radwi A., Peracha A., Ezzat A. (1998). Primary T-cell lymphoma of the urinary bladder. Am. J. Surg. Pathol..

[7] Khaitan A., Gupta N.P., Goel A., Safaya R., Kumar L. (2004). Primary non-Hodgkin’s lymphoma of urinary bladder. Report of a case and review of the literature. Urol. Int..

[8] Ohsawa M., Aozasa K., Horiuchi K., Kanamaru A. (1993). Malignant lymphoma of bladder. Report of three cases and review of the literature. Cancer.

[9] Leite K.R., Bruschini H., Camara-Lopes L.H. (2004). Primary lymphoma of the bladder. Int. Braz. J. Urol..

[10] Al-Maghrabi J., Kamel-Reid S., Jewett M., Gospodarowicz M., Wells W., Banerjee D. (2001). Primary low-grade B-cell lymphoma of mucosa-associated lymphoid tissue type arising in the urinary bladder: Report of 4 cases with molecular genetic analysis. Arch. Pathol. Lab. Med..

[11] Simpson R.H., Bridger J.E., Anthony P.P., James K.A., Jury I. (1990). Malignant lymphoma of the lower urinary tract. A clinicopathological study with review of the literature. Br. J. Urol..

[12] Bates A.W., Norton A.J., Baithun S.I. (2000). Malignant lymphoma of the urinary bladder: A clinicopathological study of 11 cases. J. Clin. Pathol..

[13] Hughes M., Morrison A., Jackson R. (2005). Primary bladder lymphoma: Management and outcome of 12 patients with a review of the literature. Leuk. Lymphoma.

[14] Sellman D.P., Simpson W.G., Klaassen Z., Jen R.P., DiBianco J.M., Reinstatler L., Li Q., Madi R., Terris M.K. (2018). Characterization and outcomes of local treatment for primary bladder lymphoma: A population-based cohort analysis. Urol. Ann..

[15] Oh K.C., Zang D.Y. (2003). Primary non-Hodgkin’s lymphoma of the bladder with bone marrow involvement. Korean J. Intern. Med..

[16] Cheson B.D., Fisher R.I., Barrington S.F., Cavalli F., Schwartz L.H., Zucca E., Lister T.A. (2014). Recommendations for initial evaluation, staging, and response assessment of Hodgkin and non-Hodgkin lymphoma: The Lugano classification. J. Clin. Oncol.: Off. J. Am. Soc. Clin. Oncol..

[17] Moons K.G., Altman D.G., Reitsma J.B., Ioannidis J.P., Macaskill P., Steyerberg E.W., Vickers A.J., Ransohoff D.F., Collins G.S. (2015). Transparent Reporting of a multivariable prediction model for Individual Prognosis or Diagnosis (TRIPOD): Explanation and elaboration. Ann. Intern. Med..

[18] Harrell F.E., Lee K.L., Mark D.B. (1996). Multivariable prognostic models: Issues in developing models, evaluating assumptions and adequacy, and measuring and reducing errors. Stat. Med..

[19] Camp R.L., Dolled-Filhart M., Rimm D.L. (2004). X-tile: A new bio-informatics tool for biomarker assessment and outcome-based cut-point optimization. Clin. Cancer Res. Off. J. Am. Assoc. Cancer Res..

[20] Vickers A.J., Elkin E.B. (2006). Decision curve analysis: A novel method for evaluating prediction models. Med. Decis. Mak. Int. J. Soc. Med. Decis. Mak..

[21] Horesh N., Horowitz N.A. (2014). Does gender matter in non-hodgkin lymphoma? Differences in epidemiology, clinical behavior, and therapy. Rambam Maimonides Med. J..

[22] Rachoń D., Myśliwska J., Suchecka-Rachon K., Wieckiewicz J., Myśliwski A. (2002). Effects of oestrogen deprivation on interleukin-6 production by peripheral blood mononuclear cells of postmenopausal women. J. Endocrinol..

[23] Fernández Aceñero M.J., Martín Rodilla C., López García-Asenjo J., Coca Menchero S., Sanz Esponera J. (1996). Primary malignant lymphoma of the bladder. Report of three cases. Pathol. Res. Pract..

[24] Balachandran V.P., Gonen M., Smith J.J., DeMatteo R.P. (2015). Nomograms in oncology: More than meets the eye. Lancet Oncol..

