# Supplementary material for: Development and Validation of Survival Nomograms in Patients with Primary Bladder Lymphoma
Source: J Clin Med. 2022 Jun 2;11(11):3188. doi: 10.3390/jcm11113188 (PMC9181374; doi:10.3390/jcm11113188)
Supplement: Supplementary file 1 [file jcm-11-03188-s001.zip › jcm-1666093-supplementary.pdf]

Development and validation of survival nomograms in patients with primary bladder lymphoma

Junyi Lin†, Jianbin Kong†, Mingli Luo, Zefeng Shen, Shuogui Fang, Jintao Hu, Zixin Xu, Wen Dong, Jian Huang*, Tianxin Lin*

Supplementary figures

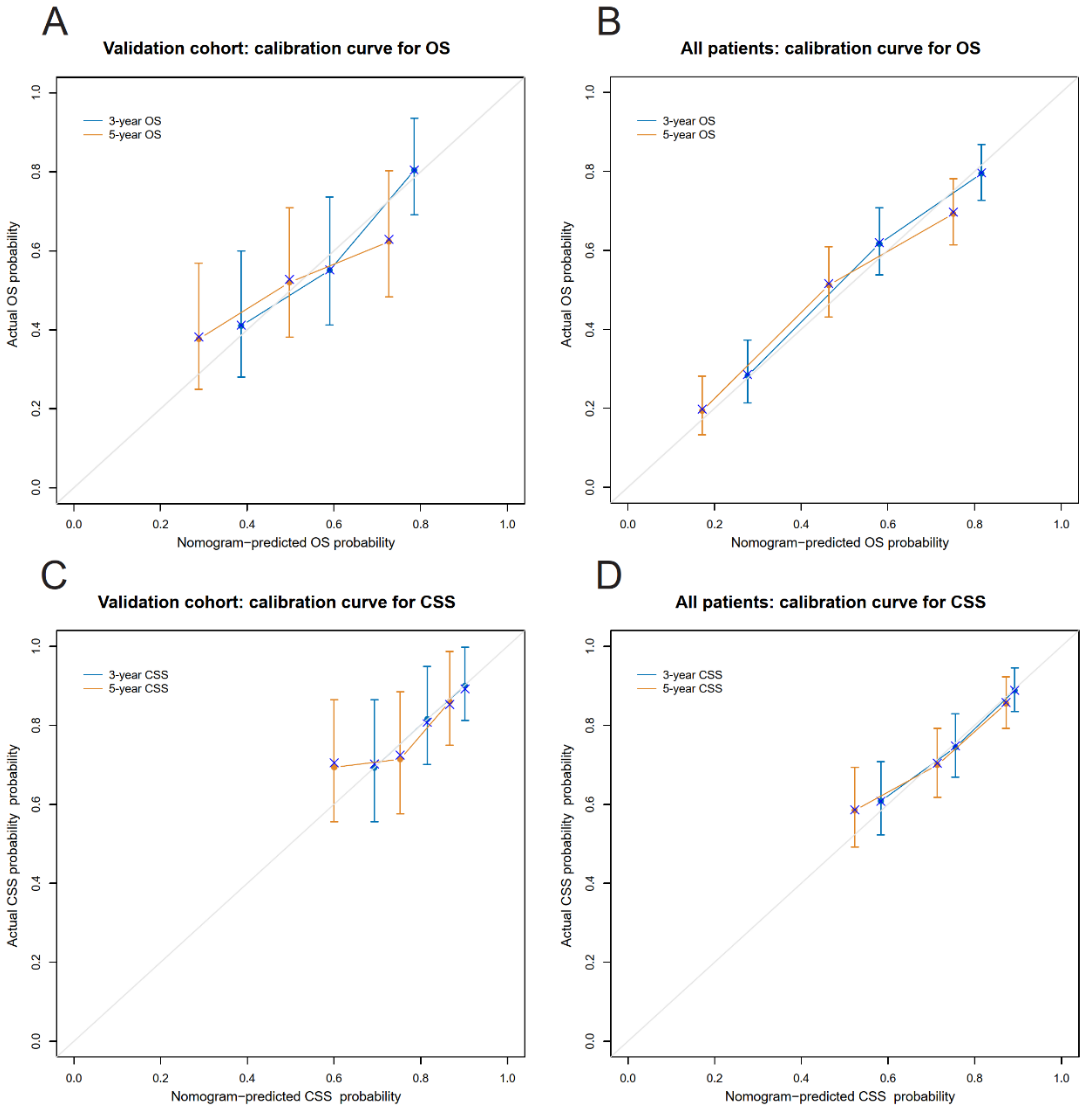

Supplementary Figure S1. Calibration curve in the validation cohort and all patients of the OS nomogram (A-B) and CSS nomogram (C-D). X-axis: nomogram predicted probability; Y-axis: actual probability. OS, overall survival; CSS, cancer-specific survival.

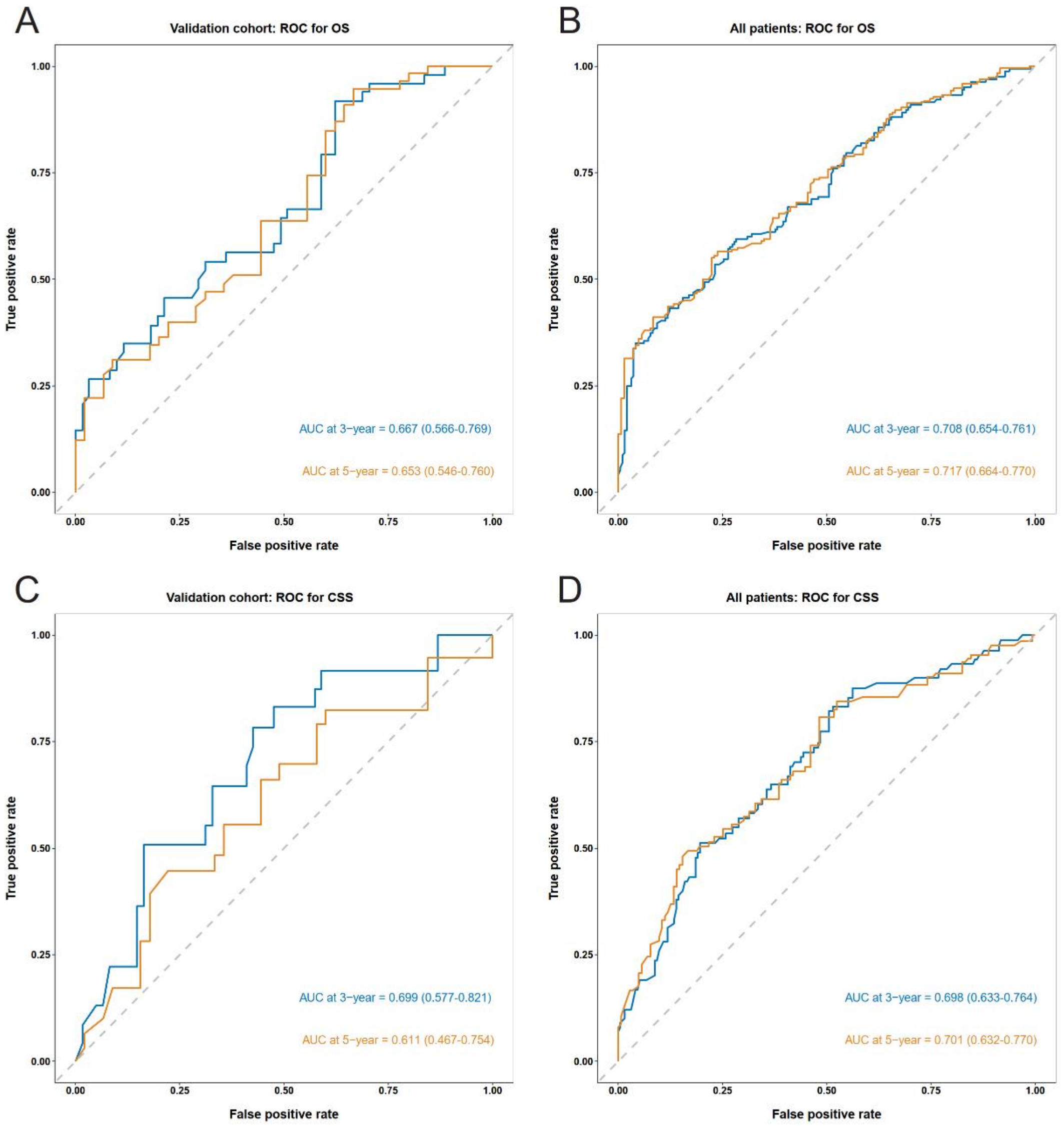

Supplementary Figure S2. The ROC in the validation cohort (A) and in all patients (B) of the OS nomogram and in the validation cohort (C) and in all patients (D) of the CSS nomogram. In the aspect of the AUC at 3-year (the blue line) and 5-year (the orange line), for the validation cohort of the OS nomogram, that was 0.667 [95% confidence interval (CI), 0.566-0.769] and 0.653 [95% confidence interval (CI), 0.546-0.760], respectively; for all patients of the OS nomogram, that was 0.708 [95% confidence interval (CI), 0.654-0.761] and 0.717 [95% confidence interval (CI), 0.664-0.770], respectively; for the validation cohort of the CSS nomogram, that was 0.699 [95% confidence interval (CI), 0.577-0.821] and 0.611 [95% confidence interval (CI), 0.467-0.754], respectively; for all patients of the CSS nomogram, that was 0.698 [95% confidence interval (CI), 0.633-0.764] and 0.701 [95% confidence interval (CI), 0.632-0.770], respectively. ROC, receiver operating characteristic curve; AUC, areas under the curve.

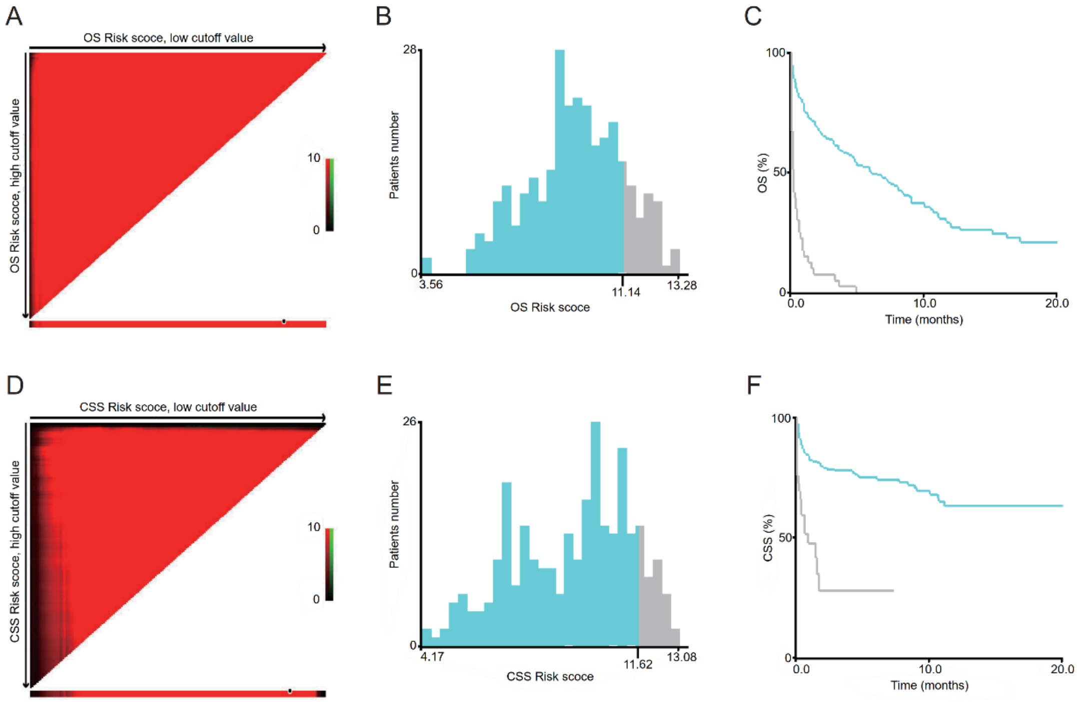

Supplementary Figure S3. The optimal cut-off value calculated by X-tile software was 11.14 for the OS nomogram score and 11.62 for the CSS nomogram score.

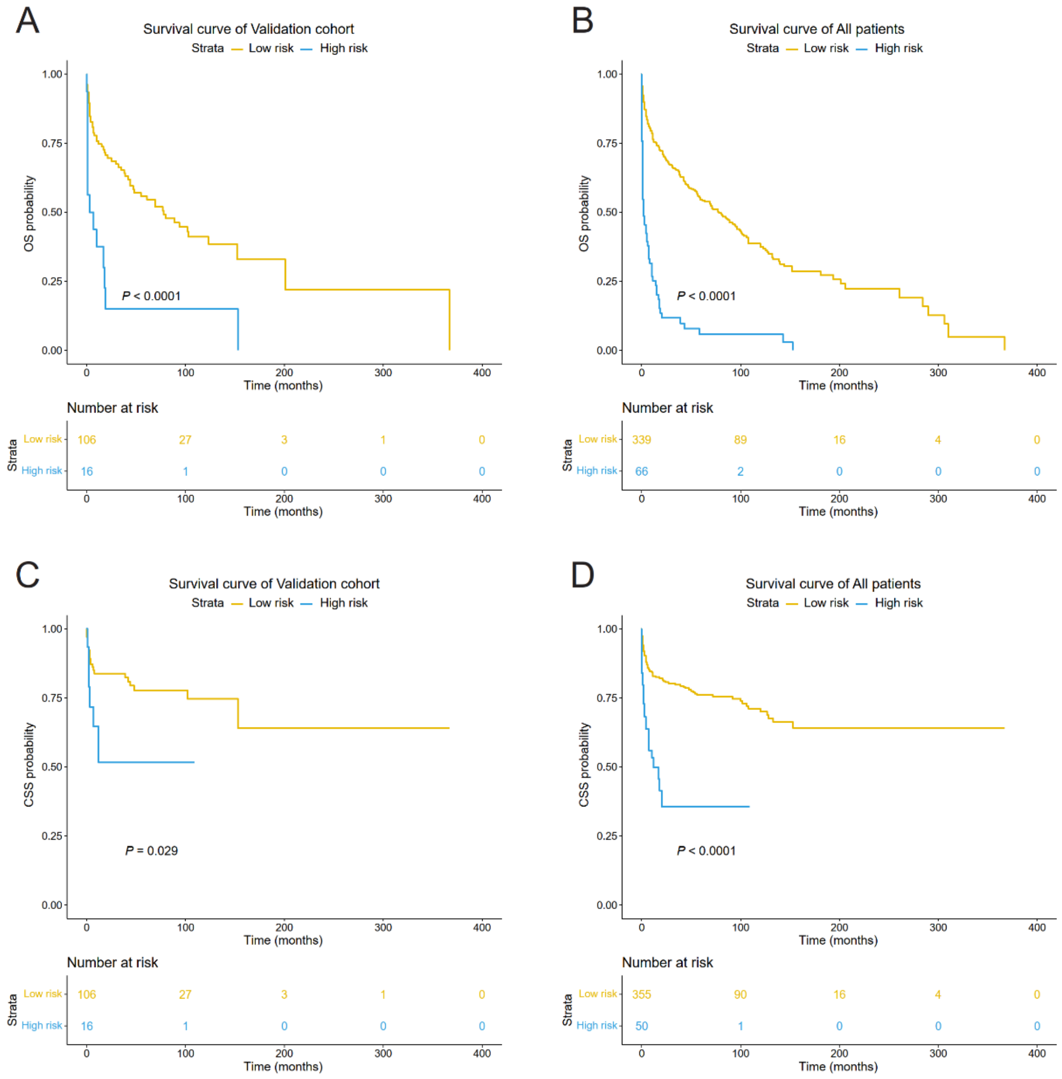

Supplementary Figure S4. The Kaplan-Meier curves in the validation cohort (A) and in all patients (B) of the OS nomogram and in the validation cohort (C) and in all patients (D) of the CSS nomogram for primary bladder lymphoma patients in the low risk group and the high risk group. Low risk of OS: nomogram score < 11.14; High risk of OS: nomogram score ≥ 11.14; Low risk of CSS: nomogram score < 11.62; High risk of OS: nomogram score ≥ 11.62; OS, overall survival; CSS, cancer-specific survival.

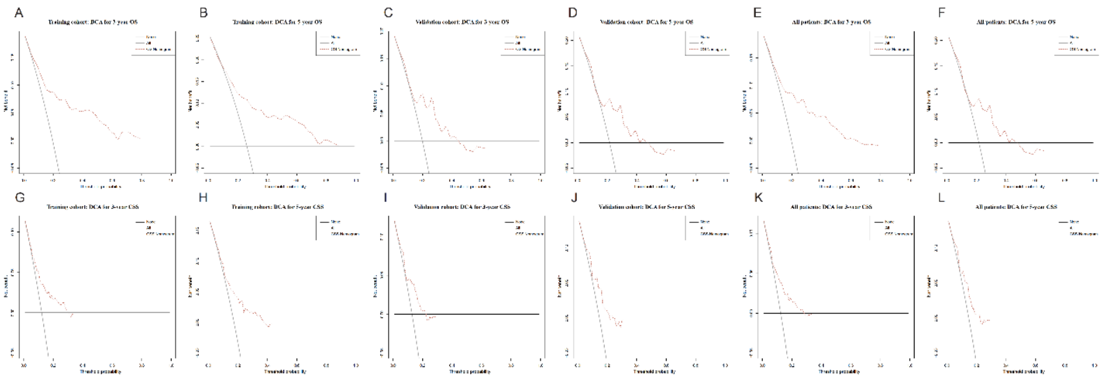

Supplementary Figure S5. DCA for OS in the training cohort (A-B), validation cohort (C-D) and in all patients (E-F), and for CSS in the training cohort (G-H), validation cohort (I-J) and in all patients (K-L). The black line represented non-treated patients, the gray line represented all treated patients, the red dotted line represented patients in the nomogram. DCA, decision curve analysis; OS, overall survival; CSS, cancer-specific survival.

Supplementary tables

Supplementary Table S1 The Cox regression coefficients of the OS nomogram.

Variable	Cox regression coefficient
**Age**	0.091
**Subtype**	
Indolent B cell NHL	Reference
Aggressive B cell NHL	2.08544
NHL-NOS	1.181182
Other/unclassified	1.057238
**Ann Arbor stage**	
Stage I	Reference
Stage II	0.2237531
Stage III	1.1559925
Stage IV	1.6095672
Unknown	0.4759728
**Radiation**	
Yes	Reference
No/Unknown	0.797671
**Chemotherapy**	
Yes	Reference
No/Unknown	0.8750378

NOS, not otherwise specified; NHL, non-Hodgkin's lymphoma.

Supplementary Table S2 The Cox regression coefficients of the CSS nomogram.

Variable	Cox regression coefficient
**Age**	0.091
**Sex**	
Female	Reference
Male	1.312299
**Subtype**	
Indolent B cell NHL	Reference
Aggressive B cell NHL	3.391435
NHL-NOS	2.648300
Other/unclassified	2.586074

NOS, not otherwise specified; NHL, non-Hodgkin's lymphoma.

Supplementary Table S3 The formula of each variable to calculate the OS risk score.

OS	Formula
**3-year OS probability**	0.003 * points ^3 + -0.091 * points ^2 + 0.774 * points + -1.043
**5-year OS probability**	0.003 * points ^3 + -0.085 * points ^2 + 0.658 * points + -0.572

OS, overall survival. Points is the sum of all Cox regression coefficients of variables included in the OS nomogram.

Supplementary Table S4 The formula of each variable to calculate the CSS risk score.

CSS	Formula
**3-year CSS probability**	0 * points ^3 + -0.005 * points ^2 + 0.047 * points + 0.850
**5-year CSS probability**	0 * points ^3 + -0.005 * points ^2 + 0.043 * points + 0.866

CSS, cancer-specific survival. Points is the sum of all Cox regression coefficients of variables included in the CSS nomogram.
